# Infections and internal medicine patients: Could a comorbidity score predict in-hospital mortality?

**DOI:** 10.1097/MD.0000000000012818

**Published:** 2018-10-19

**Authors:** Fabio Fabbian, Alfredo De Giorgi, Benedetta Boari, Elisa Misurati, Massimo Gallerani, Rosaria Cappadona, Rosario Cultrera, Roberto Manfredini, Maria A. Rodrìguez Borrego, Pablo J. Lopez-Soto

**Affiliations:** a Clinica Medica Unit, Department of Medical Sciences, University of Ferrara; b First Internal Medicine Unit, Department of Internal Medicine, General Hospital of Ferrara; c Obstetrics and Gynecology Unit, Department of Morphology, Surgery and Experimental Medicine, University of Ferrara; d Infectious Diseases University Unit, Department of Medical Sciences, University of Ferrara, Italy; e Instituto Maimónides de Investigación Biomédica de Córdoba, Universidad de Córdoba & Hospital Universitario Reina Sofía, Córdoba, Spain.

**Keywords:** comorbidity, disease management, Elixhauser, ICD-9-CM codes, infectious diseases, in-hospital mortality

## Abstract

Infectious diseases (ID) are frequently cause of internal medicine wards (IMW) admission. We aimed to evaluate risk factors for in-hospital mortality (IHM) in IMW patients with ID, and to test the usefulness of a comorbidity score (CS).

This study included ID hospital admissions between January 2013, and December 2016, recorded in the database of the local hospital. ICD-9-CM codes were selected to identify infections, development of sepsis, and to calculate a CS.

We analyzed 12,173 records, (age 64.8 ± 25.1 years, females 66.2%, sepsis 9.3%). Deceased subjects (1545, 12.7%) were older, had higher percentage of sepsis, pulmonary infections, and endocarditis. Mean value of CS was also significantly higher. At multivariate analysis, the odds ratio (OR) for sepsis (OR 5.961), endocarditis (OR 4.247), pulmonary infections (OR 1.905), other sites of infection (OR 1.671), and urinary tracts infections (OR 0.548), were independently associated with IHM. The CS (OR 1.070 per unit of increasing score), was independently associated with IHM as well. The calculated weighted risk, obtained by multiplying 1.070 for the mean score value in deceased patients, was 19.367. Receiver operating characteristic (ROC) analysis showed that CS and development of sepsis were significant predictors for IHM (area under the curve, AUC: 0.724 and 0.670, respectively).

Careful evaluation of comorbidity in internal medicine patients is nowadays matter of extreme importance in IMW patients hospitalized for ID, being IHM related to severity of disease, type and site of infection, and also to concomitant comorbidities. In these patients, a careful evaluation of CS should represent a fundamental step in the disease management.

## 1 Introduction

Increasing population life expectancy and mean age of population are the fundamental basis of growing morbidity, hospital admissions and mortality in older patients.^[1]^ World Health Organization 2016 raport shows that infectious diseases are responsible for an age specific death rate of 72.2 × 100.000 population globally, and 7.6 in Italy.^[2]^ De Buyser et al evaluated 1223 patients aged more than 81 years admitted in geriatric and internal medicine acute wards of seven Italian hospitals, and infections represented 11% of medical diagnoses, being also independent predictors of length of hospital stay.^[3]^ Our group has previously reported that in-hospital mortality (IHM) was independently associated with sepsis, urinary tract infections, female gender, and age.^[4]^ Moreover, IHM has been found to be independently associated with several comorbidities, such as renal dysfunction, myocardial infarction, stroke, and severe chronic obstructive pulmonary disease.^[5–7]^ An European study by Esteban et al^[8]^ evaluated 15,852 patients aged >18 years admitted to hospitals. The calculated incidence of sepsis was 367 cases per 100,000 adult area residents per year, and the cumulative incidence rate among patients admitted to the hospital was 4.4%. In 71% of cases the infection was community-acquired, and severe sepsis and septic shock showed an incidence rate of 104 and 31 cases per 100,000 adult area residents per year, respectively. Only 32% of severe sepsis patients received intensive care, and IHM was 12.8%, higher for severe sepsis (20.7%) and septic shock (45.7%).^[8]^ Unfortunately, data about IHM due to infectious disease in internal medicine wards (IMW) are scarce. Infections represent severe clinical conditions that could rapidly worsen and end up in IHM.

The aim of this study was to evaluate the risk factors for IHM in a large series of patients consecutively admitted to IMW for infectious diseases by using International Classification of Diseases, 9th Revision, Clinical Modification (ICD-9-CM) codes, and to test the possible usefulness of a comorbidity index, proposed by the authors and adapted for internal medicine patients, as a predictor of IHM.

## 2 Methods

### 2.1 Patient selection and eligibility

This retrospective study, conducted in agreement with the declaration of Helsinki, and approved by the local ethical committee, included all hospital admissions in internal medicine department due to infectious diseases between January 1, 2013, and December 31, 2016 recorded in the database of the General Hospital of Ferrara, Region Emilia-Romagna of Italy, and maintained by the Center for Health Statistics. The Hospital of Ferrara has been using an electronic database to store all the Discharge Hospital Sheets (DHR) of hospitalized patient, reporting name, gender, date of birth, date and department of hospital admission and discharge, vital status at discharge, length of stay, charge details, main and up to 15 accessory discharge diagnoses, based on ICD-9-CM codes. In agreement with national dispositions by law in terms of privacy, the Health authorities removed patient names, exact addresses, and other potential identifiers from the database provided for this study. The General Hospital of Ferrara is provided with 626 beds, and represents the hub center and the teaching hospital of the Province of Ferrara (around 3,50,000 inhabitants), with all facilities available, excluding only cardiothoracic surgery. Two other smaller spoke hospitals are present in the Province (around 250 beds each). The annual flow of patients by the emergency department (ED) is approximately 85,000 with significant presence of elderly subjects due to the fact that Ferrara is characterized by a high percentage of elderly subjects (26% of population aged >65 years, and near 1% >90 years). The Department of Medicine consists of four Internal Medicine units and one of Geriatrics (135 total beds, 24/24 hours and 7/7 days open to the ED admissions). Two Units of Infectious Diseases are also present (12 beds) for particular cases in which isolation or highly selected procedures are needed. About one third of all yearly hospital admissions are directed to the Department of Medicine. The great majority of medical and nursing staff is permanent, covering also festive days or holidays.

### 2.2 Data collection

All patient admitted to the Department of Medicine units with an infectious diseases diagnosis in any position were included in our study (ICD-9-CM codes: 001–139, 421, 422, 460–466, 480–487, 567, 575.0 – 575.1, 577.0, 590–599, 680–686, 711, 730). Subjects transferred to surgical departments or intensive care units (ICUs) were excluded, due to the fact that management is completely different and admission in these types of wards is decided by specialists. Readmissions are commonly seen amongst medical patients,^[9,10]^ therefore we selected to amalgamate multiple admissions for single patients as single record.

Diagnosis of sepsis was based on the following codes: “417,” “575,” and “576.” Moreover, the different infections such as pulmonary, gastrointestinal, cutaneous infections, endocarditis, and other site of infections were classified by ICD-9-CM codes. ICD-9-CM codes were also needed to calculate a modified Elixhauser Index (EI), a novel score recently proposed by our group in order to take into due account the comorbidity burden.^[11]^ The following conditions were considered for score calculation: age, gender, presence of renal failure, neurological disorders, lymphoma, solid tumor with metastasis, ischemic heart disease, congestive heart disease, coagulopathy, fluid and electrolyte disorders, liver disease, weight loss, and metastatic cancer.^[11]^ The points assigned to each condition ranged from 0 to 16, and the possible range of the score varied between 0 and 89. The risk of IHM became significant over the score of 40, overcoming the value of 60%. The score was developed using administrative data and was calculated automatically. Points assigned to different conditions in order to calculate the score for risk of IHM are reported in Table 1.

**Table 1:**
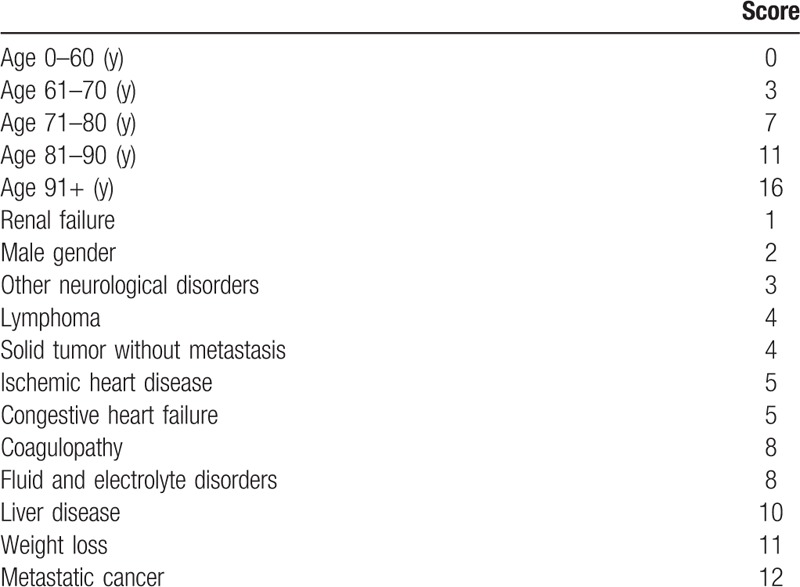
Points assigned to different conditions in order to calculate the score for risk of in-hospital mortality.

Finally, IHM related to these patients was extracted from the general database.

### 2.3 Statistical analysis

The data have been expressed as absolute numbers, percentages, and means ± SD. We compared survivors and deceased during admission, and the analysis of the variables was conducted using *χ*^*2*^, student *t* tests or Mann–Whitney *U* test, as appropriate. Moreover, in order to assess the independent parameters associated with IHM, the latter was considered as a dependent variable in a logistic regression analysis, and different infections classified as reported above and comorbidity score were considered as independent ones. Odds ratios (ORs), and their 95% confidence intervals (95% CI) were reported.

The ability of the score to discriminate between survivors and deceased was calculated using the receiver operating characteristic (ROC) curves, in which the true positive rate (sensitivity) is plotted in function of the false positive rate (100-Specificity) for different cut-off points.^[12]^ For the construction of this curve, IHM was the outcome variable and the novel score was the predictor variable. Accuracy is measured by the area under the curve (AUC). All *P*-value were 2-tailed, and a *P*-value <.05 was considered significant. SPSS 13.0 for Windows (SPSS Inc., Chicago, IL, 2004) was used for statistical analyzes.

## 3 Results

During the study period we analyzed 12,173 records corresponding to 253.6 admissions due to infectious diseases per month. Mean age was 64.8 ± 25.1 years, females were 66.2%, 9.3% developed sepsis and 12.7% of cases died. Clinical characteristics of our population are reported in Table 2. Mean value of comorbidity score was 12.3 ± 9.7, and it was significantly higher in deceased patients (18.1 ± 9.1 vs 11.4 ± 9.5, *P* < .001). Difference between survivors and deceased patients are reported in Table 3. Due to the fact that in a patient more than one infection could be diagnosed the number of infections was higher than the number of admission.

**Table 2:**
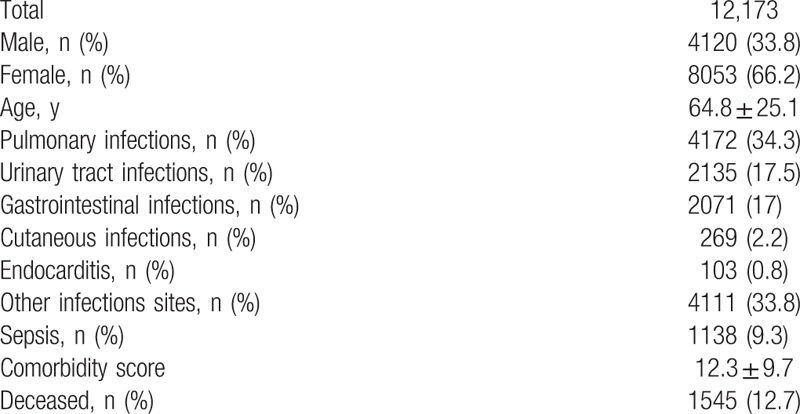
Clinical characteristics of 12,173 records related to patients admitted to internal medicine wards due to infectious diseases.

**Table 3:**
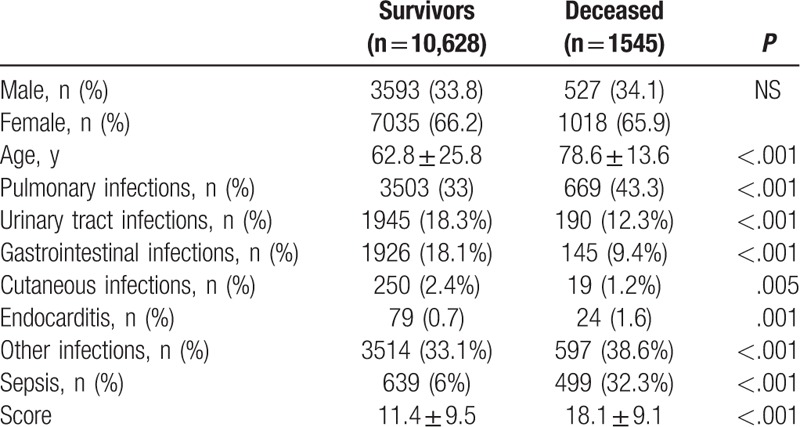
Comparison between survivors and deceased patients.

**Figure 1:**
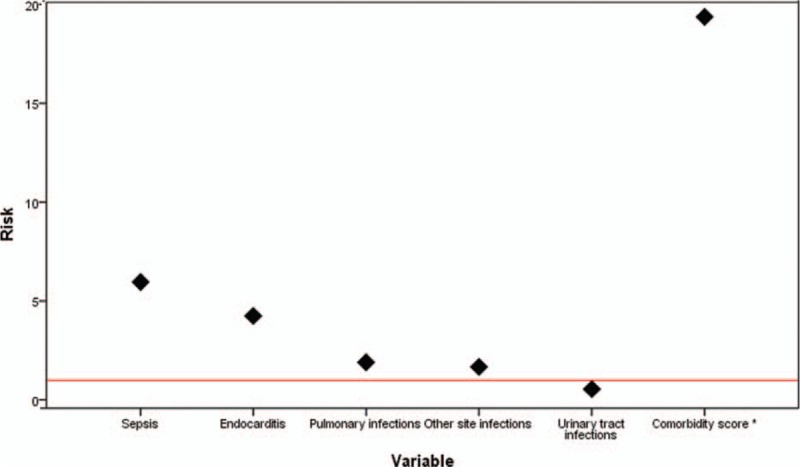
Multivariate analysis results relating association between in-hospital mortality and development of sepsis, comorbidity and different infectious diseases. ∗Weighted comorbidity score was calculated as the risk due to every unit of comorbidity score multiplied by the mean value of the score in the deceased patients.

**Figure 2:**
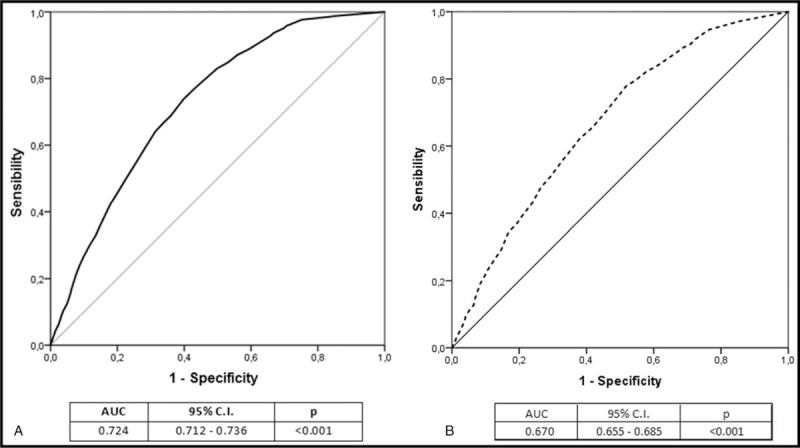
Receiver operating characteristic (ROC) curves analysis showing the ability of comorbitidy score (A) and development of sepsis (B).

At multivariate analysis, in decreasing order, sepsis (OR 5.961; 95% CI 5.187–6.850, *P* < .001), endocarditis (OR 4.247; 95% CI 2.492–7.238, *P* < .001), pulmonary infections (OR 1.905; 95% CI 1.455–2.494, *P* < .001), other sites of infection (OR 1.671; 95% CI 1.242–2.249, *P* = .001), and urinary tracts infections (UTIs) (OR 0.548; 95% CI 0.419–0.715, *P* < .001), were independently associated with IHM (Table 4). The novel comorbidity score (OR 1.070 per unit of increasing score), resulted to be independently associated with IHM as well. Thus, a weighted extrapolation obtained by multiplying 1.070 for the mean value of the score in the deceased patients, produced an weighted score of 19.367, (95% CI 19.258–19.475), much higher even than the risk due to development of sepsis (Table 4, Fig. 1). ROC analysis (Fig. 2) showed that comorbidity score and development of sepsis were significant predictors for IHM (AUC: 0.724 and 0.670, respectively).

**Table 4:**
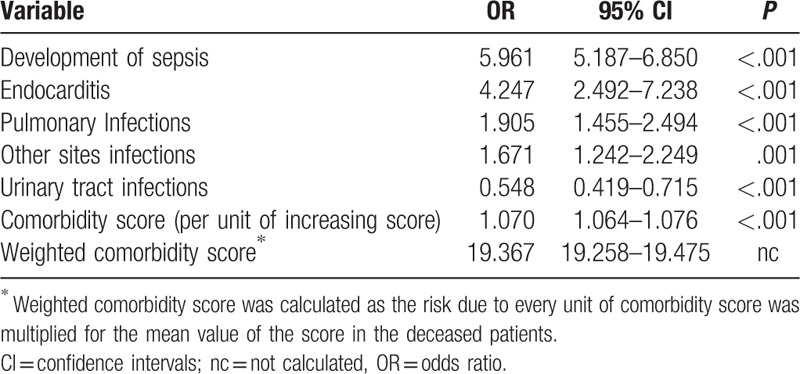
Multivariate analysis results showing variable independently associated with in-hospital mortality.

## 4 Discussion

To the best of our knowledge, this is the first Italian study conducted in IMW evaluating impact of comorbidity on IHM due to infections. In the recent past, our group has proposed and evaluated a modified version of the EI on IHM due to all causes.^[11]^ We performed a single-center retrospective study including hospital admissions for any cause in the department of internal medicine, and compared the EI with a new score obtained from EI. Prediction power of the new index was higher than the original EI. The new score included age, gender, presence of renal failure, neurological disorders, lymphoma, solid tumor with metastasis, ischemic heart disease, congestive heart disease, coagulopathy, fluid and electrolyte disorders, liver disease, weight loss, and metastatic cancer.^[11]^ With this study, we decided to test the ability of a simple comorbidity score, based on administrative data, to predict IHM in subjects admitted for infections. On one hand, development of sepsis represents the strongest risk factor for IHM in this kind of subjects. This is not surprising at all, since sepsis is a prevalent and severe medical condition and its incidence is increasing.^[13]^ On the other, comorbidity score has resulted to be a significant predictor of IHM as well. In fact, since comorbidity score gives additional OR of 1.070 per unit, the final calculated weighted risk, is even higher than that of sepsis.

Physicians should take into consideration patients with their burden of illness in their clinical practice and comorbidity appears to be crucial also in research. In order to improve prognostic assessment, patients could be assessed by every individual disease or through a number suggesting the burden of illness. Such a number could suggest multiple chronic diseases and the related prescription of multiple drugs, helping physicians in targeting intervention, and identifying subjects that could be at risk of serious adverse events. We think that the use of clinical scores could really help physicians in clinical practice. Relationship between mortality, comorbidity and infectious diseases has been considered by different authors, results are not univocal, probably due to different study design, patients’ selection, and settings. In their retrospective study, Briongos-Figuero et al^[14]^ compared 187 deaths due to infectious causes and 224 deaths due to non-infectious causes (mean age >80 years for both groups). Comorbidity was evaluated by Charlson index (CI). Dementia, cerebrovascular disease, living in nursing home and being dependent were risk factors for IHM due to infectious disease. CI was not different in the 2 groups of patients, but in this study the authors took into consideration only fatal cases.^[14]^ Yang et al^[15]^ assessed the disease burden of sepsis and evaluated the impact of CI and age as risk-adjusted hospital mortality predictors in patients with sepsis using hospital administrative database. They studied more than 300,000 hospitalized patients over 4 years, of whom 6929 (2.27%) had sepsis, and 1216 (17.5%) were admitted to ICU. The mortality rates increased consistently in patients with CI ranging from none to low, moderate and high grade for both patients with ICU admission (39.4%, 51.6%, 55.9%, and 54.3% respectively) and patients without ICU admission (6.4%, 8.7%, 17.1%, and 25.3% respectively). Logistic regression analysis showed that CI (OR 11.8) and age (OR 8.46) were independently associated with IHM.^[15]^ Since the comorbidity score used in our study just included age, we did not include this variable in the model to avoid overestimation bias. Mazzone et al^[16]^ evaluated short-term mortality in 533 septic patients treated in IMW, of whom 78 (14.6%) died during hospitalization. Cardio-cerebrovascular disease, diabetes, chronic kidney disease, respiratory disease, active cancer, or hepatic disease were the comorbid conditions considered by authors. Mortality rate was 5.5% in subjects with non-severe sepsis and 20.1% in those with severe sepsis or septic shock. Severe sepsis or septic shock (OR 4.41), active solid cancer (OR 2.14), immune system weakening (OR 2.10), and age (OR 1.03 per year) were independently related to mortality.^[16]^ Rebelo et al^[17]^ assessed independent risk factors for IHM studying retrospectively a cohort of 135 patients with bacteremia aged ≥65 years, admitted to an IMW. IHM was 22.2% and in more than 45% of subjects the cause was urinary tract infections. The main microorganisms isolated in the blood cultures were *Escherichia coli* (14.9%), Methicillin-resistant *Staphylococcus aureus* (MRSA) (12.0%), non-MRSA (11.4%), *Klebsiella pneumoniae* (9.1%), and *Enterococcus faecalis* (8.0%). IHM was independently associated, in decreasing order, with bacteremia of unknown focus (OR 8.673), chronic renal disease (OR 6.179), cognitive impairment at admission (OR 3.621), and age ≥ 85 years (OR 2.812), whereas different chronic medical conditions were equally distributed in survivors and deceased patients.^[17]^

It has to be stressed that sometimes studies are not easily comparable due to the application of scores utilized, for example, in the EDs or ICUs, often requiring data unavailable in IMW. In fact, in the ED settings different clinical scores were tested in order to identify the high risk infected patients, but such scores showed heterogeneous ability to predict hard outcomes such as 28-day mortality or IHM, due to different clinical manifestations related to sites of infection, comorbidity, and underlying microorganisms. The MEDS (Mortality in Emergency Department Sepsis) score^[18]^ was one of the first clinical scores for emergency settings used to predict the 28-day mortality risk, and its AUC was 0.82. A few years later, Sankoff et al^[19]^ tried to propose a modified MEDS score (mMEDS), removing the lack of neutrophil band counting, but final predictivity was the same (AUC: 0.82). Ghanem-Zoubi et al^[20]^ evaluated the ability of either MEDS and another 3 scoring systems: modified early warning score (MEWS), simple clinical score (SCS), and rapid emergency medicine score (REMS). In order to stratify for utilization management, performance assessment, and clinical research, they studied 28-day and overall IHM, and 30- and 60-day mortality in 1072 patients meeting sepsis criteria and admitted to IMW. The AUC for each scoring system was 0.73 to 0.75 for MEDS, 0.65 to 0.70 for MEWS, 0.76 to 0.79 for SCS, and 0.74 to 0.79 for REMS.^[20]^ All these scores, however, were characterized by collection of different clinical information, such as heart rate, temperature, systolic blood pressure, but information on comorbidity was scarce.

Again, different score systems for severity of illness have been validated as tools to predict the risk of death in ICU patients, but a systematic review performed by Calle et al^[21]^ did not provide enough information to assess the accuracy of the prognostic models in patients with suspected infection admitted to the ED and hospital ward. More recently, Chen et al^[22]^ compared the prognostic performance of MEDS and other 2 clinical scores, the Predisposition, Infection, Response and Organ dysfunction (PIRO) staging system, and the Acute Physiology and Chronic Health Evaluation (APACHE II) scores in patients admitted to ICU. All 3 systems were independent predictors of 28-day mortality with similar AUC values. The AUC of PIRO was 0.889 for ICU admission, 0.817 for multiple organ dysfunction.^[22]^

In this study we found that UTIs had protective effect for IHM. In a previous investigation,^[4]^ we analyzed retrospectively diagnosis of UTIs based on ICD-9-CM codes and IHM, 5 percent of cases developed sepsis and 3.7% had a fatal outcome. The latter percentage was less than one third of the mortality recorded in this study; moreover, development of sepsis was independently related to IHM. It could be that physicians early recognize UTIs, especially in subjects with high comorbidity, suggesting the beginning of specific treatment.

## 5 Limitations and strengths

We are aware of several limitations of our study. First, this is a cross observational retrospective study based on ICD-9-CM codes, characterized by a low sensitivity and specificity.^[23]^ Data based on ICD-9-CM codes are characterized by low sensitivity and specificity, and do not provide information on reason for admission, specific cause of death, disease severity, microbial blood isolation, functional status, intensity care level, including aggressive therapy, and/or devices use. We are aware that description of the micro-orgasm that caused the infection would be interesting, especially in differentiating community and health related infections, however due to our study design this information is lacking.

Recently it has been developed a clinical model for predicting IHM in unselected acute medical admissions including age, body mass index, mean arterial pressure on admission, prior admission within 3 months, heart failure, active malignancy, and chronic use of statins, and antiplatelet agents.^[24]^ Moreover mortality risk of pediatric sepsis patients was assessed extracting data of admission to the pediatric ICU. Authors identified 6 variables associated with IHM such as brain natriuretic peptide, albumin, total bilirubin, d-dimer, lactate levels, and mechanical ventilation.^[25]^ Many of the above mentioned parameters were not evaluated in our study due to the fact that they were not available in the analyzed database based on ICD-9-CM.

Lack of specific clinical information, effect of administrative use (ie, reimbursement), possible misclassification of outcomes, and difficulties in controlling confounding factors can be considered evident disadvantages.^[26]^ Second, this is a single center study, with the great majority of Caucasian ethnicity only, and results could be different if several hospitals or different ethnic groups would be included. Generalization of a prognostic model can be limited by a given historical period, geographic location, methodological approach, or follow-up interval.^[27]^ Moreover, the model tested in the present study should be prospectively validated. Third, we had no information about out-of-hospital mortality.

However, some possible strength may also be considered. To the best of our knowledge, this is the first study conducted in IMW in Italy. The population size is not negligible, and IHM represents a hard outcome. Again, analysis of administrative data have also some pros: it allows analysis to wide population coverage and large sample size, long observation periods, low costs, and possibility to link several different sources of information. Moreover, there is also convincing evidence that use of administrative data makes possible to predict hospital admissions and complications.^[28]^

## 6 Conclusions

Careful evaluation of comorbidity in internal medicine patients is nowadays matter of extreme importance.^[29–32]^ This seems to be true also in the case of patients hospitalized to IMW for infectious diseases. In fact, the results of this study show that IHM depends, in addition to type and site of infection, also on concomitant comorbidities. Thus, in this kind of patients, especially elderly, a careful evaluation of comorbidity status by means of a simple score could represent a fundamental step in the disease management.

## Acknowledgments

We thank Franco Guerzoni and Nicola Napoli, Center for Health Statistics, Hospital of Ferrara, for their precious and valuable collaboration.

This research represents the prosecution of a project presented during the meeting of Italian Society of Internal Medicine (SIMI) in October 2016 and awarded with the Alberto Malliani Prize. The authors deeply thank SIMI for such recognition.

## Author contributions

FF and ADG had the primary idea and wrote the article, ADG, BB, RC collected the data, FF, ADG, BB, EM, RC, PJLS and MARB analyzed data and contributed to the discussion, RC and EM warranted infectious disease expertise and supervision, FF, ADG, MG, RC, RC, MARB, RM, and PJLS participated in writing discussion and conclusions, FF, ADG, MARB, RM, and PJLS reviewed/edited the article and made final supervision. All authors have made a substantial, direct, intellectual contribution to the work.

**Conceptualization:** Fabio Fabbian, Alfredo De Giorgi, Massimo Gallerani, Maria A. Rodrìguez Borrego, Roberto Manfredini, Pablo J. Lopez-Soto.

**Data curation:** Fabio Fabbian, Alfredo De Giorgi, Benedetta Boari, Elisa Misurati, Massimo Gallerani, Rosaria Cappadona, Rosario Cultrera, Maria A. Rodrìguez Borrego, Roberto Manfredini.

**Formal analysis:** Fabio Fabbian, Alfredo De Giorgi, Massimo Gallerani, Rosario Cultrera.

**Investigation:** Benedetta Boari, Elisa Misurati, Rosaria Cappadona, Rosario Cultrera, Maria A. Rodrìguez Borrego.

**Methodology:** Fabio Fabbian, Alfredo De Giorgi.

**Supervision:** Fabio Fabbian, Maria A. Rodrìguez Borrego, Roberto Manfredini.

**Validation:** Fabio Fabbian, Alfredo De Giorgi, Benedetta Boari, Elisa Misurati, Rosaria Cappadona, Rosario Cultrera, Roberto Manfredini, Pablo J. Lopez-Soto.

**Visualization:** Alfredo De Giorgi, Benedetta Boari, Elisa Misurati, Massimo Gallerani, Rosaria Cappadona, Pablo J. Lopez-Soto.

**Writing – original draft:** Fabio Fabbian, Alfredo De Giorgi, Maria A. Rodrìguez Borrego.

**Writing – review & editing:** Fabio Fabbian, Roberto Manfredini, Pablo J. Lopez-Soto.

Fabio Fabbian orcid: 0000-0001-5189-3695.
